# Priming effects in reading words with vertically and horizontally mirrored letters

**DOI:** 10.1177/17470218221141076

**Published:** 2023-01-18

**Authors:** Katharina Pittrich, Sascha Schroeder

**Affiliations:** Department of Educational Psychology, University of Göttingen, Göttingen, Germany

**Keywords:** Mirror letters, reading, visual word recognition, masked priming

## Abstract

We conducted two masked priming experiments to examine how the orthographic system processes words with mirrored letters. In both experiments, four different primes were used: an identity prime, an unrelated control prime, and two mirror-primes in which letters were either mirrored at their vertical or horizontal axis. Task was varied between experiments: In Experiment 1, we used a lexical decision task, and in Experiment 2, we used a cross-case same–different match task. We expected to see priming effects in both mirror-conditions with stronger effects in the vertically than in the horizontally mirrored letters. In the lexical decision task, we observed only vertical priming effects for words, whereas in the same–different task, priming effects were present in both mirror-conditions and for both words and non-words. We discuss the implications of our findings for extant models of orthographic processing.

Between the age of 5 and 6, many children pass through a transitional stage at which they spontaneously mirror-write their name. This may occur because pre-existing reading–related functions are recruited (see the Functional Coordination Framework, Lachmann & Geyer, 2003; Lachmann & van Leeuwen, 2008) or because novel functions utilise and “recycle” an existing brain circuitry (see the neuronal recycling hypothesis, Dehaene, 2004). A key feature of the visual system is that it exhibits mirror-generalisation (Bornstein et al., 1978), that is, visual objects are typically not only recognised in their original form, but also when a corresponding mirror image is presented. Mirror-image generalisation has been found for left–right (vertical) mirror-image reversals in both humans (Standing et al., 1970) and non-human primates (Logothetis & Pauls, 1995). These reversals are not just about viewpoint invariant object recognition as this would result in invariant perception of any orientation (Gregory & McCloskey, 2010). Rather, it applies to vertically mirrored stimuli (e.g., b vs. d) but not to stimuli which are rotated 180° clockwise (b vs. q) (Fernandes et al., 2021). Lachmann (2002) proposed the term *symmetry-generalisation*, suggesting that generalising visual input (both vertically and horizontally) warrants an advantage for visual processing of objects (and scenes), whereas the special role of vertical symmetry might be rooted in the symmetrical organisation of the cortex (or vice versa). Mirror-image discrimination is particularly important during visual recognition of alphanumeric symbols, and in particular, of letters of a script which comprises reversible graphs. Thus, mirror-image generalisation is thought to be suppressed (Lachmann, 2002), progressively unlearned (Dehaene et al., 2005, 2010; Pegado et al., 2011; Pegado, Nakamura, & Hannagan, 2014) or inhibited (Ahr et al., 2016; Duñabeitia et al., 2011; Perea et al., 2011) with reading acquisition and letters are thought to be processed mainly analytically, that is, based on their composite features, but not holistically any more (Fernandes et al., 2014; Lachmann & van Leeuwen, 2014). It is still debated whether fluent adult readers process normal and mirrored texts differently during early, automatic stages of visual word recognition. A study using event-related potentials (ERPs) by Duñabeitia et al. (2011) reported a mirror-priming effect that was similar to the identity priming effect in literate adults (at ∼250 ms). However, Pegado et al. (2014a) found early differences in processing of normal and vertically presented script (at ∼100–150 ms) in the left occipito-temporal region. Furthermore, it is unclear whether previously observed effects of mirroring generalise to horizontally mirrored text. To address this question, we conducted two masked priming experiments. In both experiments, targets were preceded by primes in which letters were either vertically or horizontally mirrored. In the first experiment, participants performed a lexical decision task, and in the second experiment, they performed a cross-case same–different match task. We expected to see priming effects in both the vertical and the horizontal condition, with stronger priming effects for vertically mirrored letters. Furthermore, we assumed that mirror-priming effects are pre-lexical and can be observed for both words and non-words.

## Introduction

For most non-artefacts (i.e., plants and animals, rocks and mountains, rivers and seas, the human body), the ability to distinguish an object from its vertical mirror-image is irrelevant because they are largely unaltered by left–right reflection (Corballis & Beale, 1976). It is, however, necessary during the visual recognition of letters. The perception of mirror-images is intimately related to the perception of symmetry which has been found to be anisotropic, that is, favouring vertical over horizontal symmetry (Corballis & Roldan, 1975; Julesz, 1971; Morin, 2018; Rossi-Arnaud et al., 2012; van der Helm & van der Helm, 2014), which is most likely a result of visual experience (Cattaneo et al., 2010). Similarly, research on mirror-image discrimination in different species and humans has shown that the inability to discriminate mirror-images applies in particular to vertical reversals (Corballis & Beale, 1976; Dehaene, 2004; Rollenhagen & Olson, 2000). In line with this hypothesis, adults have shown to reveal stronger difficulties in mirror-image discrimination tasks of vertically compared with horizontally mirrored visual shapes (Gregory & McCloskey, 2010; Sekuler & Houlihan, 1968; Sekuler & Pierce, 1973) but this perceptual bias seems to depend also on the salience of object features (Gregory & McCloskey, 2010).

Corballis and Beale (1976) have argued that implicit vertical mirror-image reversals occur because the structural formation of memory traces in the brain is symmetrised through inter-hemispheric mirror-image reversal. In other words, the brain per default encodes visual input together with its vertical mirror-image reversal to generalise from particular learned experiences to their mirror-images. According to the neuronal recycling hypothesis (Dehaene, 2004), cortical regions which were initially used for visual object recognition are recycled and re-used for the processing of written language. As a result, pre-literate children process words like other visual objects and, thus, letters and words are subjected to be mirror-reversed. Mirror-confusions in reading and writing have intrigued researchers since the beginning of the 20th century (Orton, 1925). Children who are not yet proficient readers (i.e., between the age of 5 and 10) tend to treat mirror-images of letters and sometimes entire words (i.e. 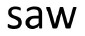 and 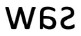) as equivalent.

These involuntary mirror-confusions vanish when reading becomes more automatised. Fernandes et al. (2016) compared 5- to 7-year-old pre-schoolers and first graders in a same–different match task in which letters of the Latin alphabet had to be distinguished based on orientation (orientation-based judgements) or judged the same based on shape (shape-based judgements). Participants saw both reversible and non-reversible letters which were vertically mirrored (i.e., b vs. d) and rotated clockwise by 180° (i.e., b vs. q). Judging two letters as the same based on shape requires participants to respond same to identical, mirror-image, and plane-rotation trials and thus the task is facilitated by mirror-image generalisation. Results showed that on orientation-based (explicit) judgements, first graders outperformed pre-schoolers. By contrast, on shape-based (automatic) judgements, first graders were slower for mirrored than identical pairs, and even slower than pre-schoolers. Only first graders revealed worse shape-based judgements for mirrored and rotated pairs for reversible compared with non-reversible letters. Together, these results provide evidence that reversible letters produce interference only once children have learned how to read. These interferences of reversible letters occur because readers find it difficult to ignore orientation contrasts relevant to letters. It is also consistent with the idea that letters are mainly processed analytically after reading acquisition, while words might still be processed holistically (Wong et al., 2011).

The influence of mirror-image generalisation on reading processes also seems to be script-specific. For example, Winskel and Perea (2022) used a same-different matching task in which a false font, faces, and letter strings were reversed (e.g., obli vs. idlo). Adult participants were required to make a same response to both identical and mirror-image stimuli. Readers of Thai (a writing system which does not comprise reversible letters) were better at recognising mirror-image pairs as the same object than English readers, indicating that the suppression of mirror-image generalisation is enhanced by learning to read in a script which comprises reversible letters.

In another recent study, Fernandes et al. (2021) used the same task as Fernandes et al. (2016) to compare the ability to discriminate mirror-images of letters in adult readers of two different writing systems: the alphabetical writing system and Tamil, which, like Thai, does not comprise reversible letters. Illiterates, monolingual Tamil literates, and Tamil–English bilinguals performed a speeded same–different match task in which letters of the Latin alphabet had to be distinguished based on orientation or judged the same based on shape. Participants saw both reversible and non-reversible letters which were vertically mirrored (i.e., b vs. d) and rotated clockwise by 180° (i.e., b vs. q). Results showed that only Tamil–English bilinguals exhibited (task-irrelevant) *automatic* mirror-image discrimination: when comparing performance in shape-based judgements, bilinguals revealed a mirror-processing cost for reversible compared with non-reversible letters, whereas monolingual Tamil speakers did not. Furthermore, only biliterates showed a reduced disadvantage on orientation over shape-based judgements for mirrored but not rotated letters, indicating that learning to read a script with reversible letters enhances the suppression of mirror-image generalisation.

Until present, however, it is less clear whether automatic mirror processing keeps affecting the early, automatic stages of the visual word recognition process in fluent adult readers. These early orthographic processes can be examined using the masked priming paradigm (Forster & Davis, 1984). In masked priming, participants are presented very briefly (i.e., 50 ms) with a prime which is followed by a target word. The prime is presented too briefly to be consciously perceived by the reader. To ensure that effects of the prime on target word recognition are based on orthographic rather than visual overlap, primes are presented in lowercase and targets in uppercase letters. Depending on the relationship between prime and target, priming effects can be either inhibitory or facilitatory. Identity priming (table–TABLE) usually produces strong facilitatory effects while unrelated control primes (house–TABLE) slow down target processing.

Only a few studies used the masked priming paradigm to investigate processing of mirrored letters. For example, Perea et al. (2011) used a masked priming lexical decision task to compare the recognition of target words which either comprised a reversible letter (e.g., i**d**ea–I**D**EA) or only non-reversible letters (e.g., a**r**ena–A**R**ENA). Primes were presented in three different conditions: Either the critical letter was presented normally (identity condition), vertically mirrored (mirror-condition) or it was replaced by an unrelated control letter (e.g., i**l**ea–IDEA) (control condition Experiment 1) or a 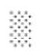 character as a missing letter (control condition Experiment 2). Results showed that primes which comprised a reversible mirrored letter slowed down target word processing more than control primes. By contrast, primes which comprised a non-reversible mirrored letter did not slow down target word processing compared with the control condition, indicating that mirror-primes with reversible letters produce mirror-interference effects, whereas mirror-primes with non-reversible letters do not. Perea and colleagues concluded that the suppression of mirror-image generalisation is applied selectively only to those letters that are reversible. Soares et al. (2019) replicated these effects in a (go/no-go) masked priming lexical decision task. In addition, they found that mirror-interference effects are mediated by the letter’s left-right orientation (i.e., whether the letter faces to the right like *b* or to the left like *d*).

A second line of studies has used masked mirror-priming to investigate potential facilitatory effects of mirroring for non-reversible letters. The underlying hypothesis is that mirrored letters also activate their mirror-image because, as suggested by Corballis and Beale (1976), visual input is per default encoded together with its mirror-image reversal. As a consequence, processing of a non-reversible letter should be facilitated by a mirror-prime compared with an unrelated control letter.

Based on this reasoning, Duñabeitia et al. (2011) used a masked priming go/no go semantic categorization task in which all internal letters of the prime were vertically mirrored while initial and final letters were presented in their normal position. Words comprised only non-reversible letters to avoid the aforedescribed mirror-interference effects. In the mirror-condition, internal letters were mirrored vertically, whereas in the control condition, the critical internal letters were replaced by other mirrored letters. Duñabeitia and colleagues found that mirror-priming effects on early electrophysiological brain responses were as pronounced as the effects evoked by the identity prime. Furthermore, they found similar results in a second experiment in which vertical mirror-primes consisted of an entirely mirrored word rather than a word with individually mirrored letters. However, they did not investigate letters which were horizontally mirrored.

Similarly, Winskel and Perea (2018) conducted two same–different masked priming experiments in which native English readers were presented with four-letter word pairs. In their critical mirror-condition, the two internal letters (all non-reversible and non-symmetrical letters) were mirrored vertically, whereas the initial and final letters were presented in their normal form. In the control condition, the two internal letters were replaced by two different letters which were also mirrored vertically. Winskel and Perea found facilitatory mirror-priming effects which were, however, less pronounced than the identity prime effect. In a replication of this experiment with native readers of Thai, they found that mirror-priming effects were similarly strong as identity priming effects. Again, the authors did only investigate the effects of vertical mirroring. In addition, they did not include non-words in their experiment.

In a recent masked priming study, Brossette et al. (2022) conducted three experiments to examine mirror-priming effects for primes with non-reversible letters which were all mirrored vertically. In addition they manipulated word frequency. In the first two experiments they compared a conventional and a sandwich masked priming paradigm and used digit string primes in the control condition. They found mirror-priming effects only in the sandwich paradigm and only for high-frequency words. In the third experiment, they replaced digit string controls by letter-string controls and used a new material set to address concerns regarding prime-target visual overlap. They found mirror-priming effects for non-reversible letters that produced additive effects with word frequency, indicating that the mirror-priming effect operates on an early, automatic stage of orthographic processing. Importantly, unlike Duñabeitia et al. (2011), Brossette and colleagues found that mirror-priming effects were less pronounced than the identity priming effect (graded mirror-priming effects), and hence, the authors argue that complete preservation of mirror-image generalisation is unlikely to explain their results.

Together, the aforementioned studies show that vertical mirror-primes can affect visual word recognition and the observed effects can be inhibitory or facilitatory depending on whether the letter is reversible or non-reversible. There are two main questions which remain unresolved. First, we do not know whether the observed mirror-priming effects generalise to primes with up-down (horizontally) mirrored letters. It is plausible to believe that horizontal mirroring would produce similar effects because there is a substantial body of evidence showing that horizontal mirror-image confusions are a common phenomenon in babies (Bornstein et al., 1978), 4-year-old children (Huttenlocher, 1967), first-grade children (Sekuler & Pierce, 1973), adults with a specific reading impairment (McCloskey et al., 1995; McCloskey & Rapp, 2000) and adults without a specific reading impairment (Gregory & McCloskey, 2010; Sekuler & Houlihan, 1968; Sekuler & Pierce, 1973). The presence of a common difficulty in distinguishing both horizontal and vertical mirror-image reversals suggests that both types of mirroring may be a residual aspect of mirror-image generalisation.

Second, it is not clear whether mirror-priming effects only occur in words or also in non-words. Thus, we do not know whether mirror-priming effects are generated during lexical or pre-lexical processing. The reason for this is that previous studies have used either a semantic categorization task or a same–different match task in which only words have been included. In addition, those studies that have used non-words in a lexical decision paradigm used a go–no go task in which no responses for non-words are recorded. This is unfortunate because there are strong theoretical reasons to believe that mirror-priming effects should be located on the pre-lexical level. If mirror-image generalisation is not completely unlearned but rather selectively suppressed or inhibited for words and letters, then it is plausible to believe that at initial stages of processing, words are processed as other visual objects and therefore being subject to mirror-generalisation (Duñabeitia et al., 2011; Poldrack et al., 1998) or symmetry-generalisation (Lachmann, 2002). An early stage at which the system does not discriminate between letters in their normal orientation and mirrored letters would affect processes located at the interface between purely visual processing and pre-orthographic assembly of the letters and words (Duñabeitia et al., 2011).

### The present study

To address these questions, we conducted two masked priming experiments using the same set of stimuli. In Experiment 1, participants performed a lexical decision task, while in Experiment 2 they performed a cross-case same–different match task (see Fernandes et al., 2022, for a similar approach). In each experiment, we included both horizontal (e.g., 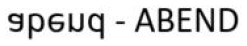) and vertical (e.g., 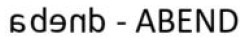) mirror-primes which were entirely written in mirrored letters. We expected priming effects for both types of mirroring, but also that vertical mirror-piming effects might be stronger. This prediction is in line with Corballis and Beale (1976), Dehaene (2004), and Rollenhagen and Olson (2000), who suggest that the perceptual system is particularly biased towards implicit vertical mirror-image generalisation.

Our predictions on the cognitive locus of mirror-priming effects are less clear. If mirror-priming effects are lexical, we would expect to see priming effects only for words, but not non-words (i.e., because non-words are not represented in the mental lexicon). If, by contrast, mirror-priming effects are pre-lexical, they should be observable for both words and non-words.

Our study differs from previous experiments by the fact that not only single, individual letters in the prime have been mirrored, but all letters at the same time. Because target words included both reversible (*b*, *d*, etc.) and non-reversible letters (*r, k*, etc.), we checked whether mirror-priming effects were moderated by a mirror-sensitivity index of a letter-string which quantifies the number of reversible, non-reversible and symmetrical letters within a string.

## Experiment 1

### Methods

All materials, data, and analysis scripts can be found in the Open Science Framework, at the following URL: https://osf.io/7uvw2/?view_only=57ccb027657e4a6989a3d46e97a14184.

#### Participants

We recruited adult participants via the recruitment database of the University of Göttingen for an online experiment in which 30 adults (age: *M* = 24.5, *SE* = 5.6 years; 25 females) participated for course credit. All participants were German native speakers and had normal or corrected-to-normal vision. The study was approved by the ethics committee of the University of Göttingen. At the beginning of the study, participants provided informed consent electronically.

#### Materials

We selected 192 nouns from the Digital Dictionary of the German language (DWDS) (Geyken, 2007) that served as target words in the lexical decision task. All words had a normalised lemma frequency >35. In addition, we created 192 non-words by substituting one or two letters of a different set of existing words. All words were nouns and comprised only one stem that was four to seven letters long. Words had a bigram frequency of *M* = 28.17 (*SD* = 4.81) and non-words had a bigram frequency of *M* = 28.84 (*SD* = 4.85).

**Figure 1.**
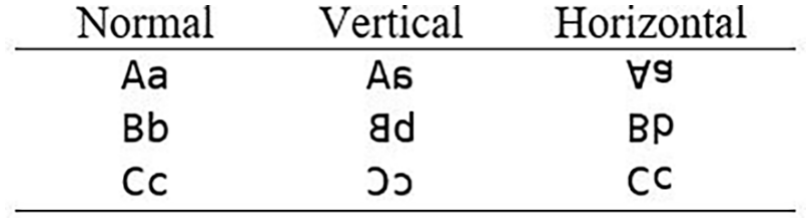
Example letters in each of the customised mirror-fonts.

Each word and non-word target was preceded by a different prime in each of four prime conditions. Targets were always presented in uppercase, while primes were always presented in lowercase letters. In the identity condition, the prime was the target word itself (vater–VATER). In the vertical mirror-condition, letters were mirrored around their vertical mirror-axis, whereas in the horizontal mirror-condition letters were mirrored around their horizontal mirror-axis. For the two mirror-prime conditions, we used the open source software Font Forge to create new customised vertical and horizontal mirror-fonts. An example of the font used in the Normal, Vertical, and Horizontal condition is shown in Figure 1.

In the control condition, the prime was an unrelated word (halle–LICHT) or non-word (stend–PFURD) and the control primes were created by reassigning one of the words to a different target. This procedure might create some overlap between control prime and target. To check this, we computed an overlap score, which counted the number of letters shared by the control and the target in the same position. For most targets, there was no (58%) or only one (34%) letter in the same position in the control prime as in the target. Two items were discarded from all analyses because more than three letters were shared between prime and target. To control for remaining differences, we included the overlap score as a control variable in all analyses.

To avoid repetition of the targets within the experiment, we used a Latin square design with four lists, such that each participant saw each target once in one of the four prime conditions. Across participants, each target was presented in combination with all four primes. Each participant saw 192 words and 192 non-words with 48 words and 48 non-words in each of the four prime conditions.

As a consequence, the present experiments are reasonably well-powered and the number of overall observations in each cell (30 × 48 = 1440) is close to the number of data points recommended by Brysbaert and Stevens (2018) to observe a 16 ms orthographic priming effect with a power of 1 − β = .8 at α = .05.

#### Mirror-sensitivity index

As elaborated above, all letters in a word were mirrored in our study, irrespective of whether they were reversible or not. To investigate whether priming effects were moderated by the number of reversible and non-reversible letters within a prime, we computed an overall *mirror-sensitivity* score which summarises how many letters in a prime are reversible, non-reversible, or symmetrical. The score quantifies how easy the letters in a word can be confused with other letters when being mirrored. The score was computed only for the primes and, thus, only for the lowercase letters.

Values were computed for each target and each mirror-condition separately in the following way: In a first step, each letter was coded using a (−1, 0, 1) coding scheme according to its mirror-sensitivity. The code 1 was assigned to reversible letters (e.g., *b*, *d*, *p*, *q*) while the code 0 was given to non-reversible letters (e.g., *r*, *c*, *g*, *k*). In addition, the code −1 was used for symmetrical letters (e.g., *x*, *o*, *l*), which stay invariant during mirroring.

As some letters are confusable with other letters when mirrored horizontally, but not vertically (e.g., *f/t*), or letters may be symmetrical around their horizontal but not their vertical symmetry axis, the coding was done independently for the vertical and the horizontal condition (e.g., the letter “c” was coded as symmetrical for the horizontal score, but not the vertical score). Similarly, the letters f and t were coded as confusable for the horizontal score, but not the vertical score.

In a next step, the mean mirror-sensitivity of a word was computed by averaging the codes of words’ component letters and by averaging the vertical and the horizontal score. Higher mirror-sensitivity scores thus indicate a higher proportion of reversible letters and a lower proportion of symmetrical letters within a word. For example, the word “abend” would be coded as (0, 1, 0, 0, 1) in the vertical condition and with (0, 1, 0, 1, 1) in the horizontal condition, leading to a mirror-sensitivity score of 0.4 in the vertical and of 0.6 in the horizontal condition.

Thus, words with a high mirror-sensitivity score are more likely to be confused when being mirrored as they comprise a larger proportion of reversible letters. By contrast, words with a low mirror-sensitivity score are unlikely to be confused when being mirrored because they comprise a lower proportion reversible letters and/or a higher proportion of symmetrical letters.

The mirror scores of word and non-word primes are provided in Table 1. A two-sample *t* test showed that the horizontal mirror scores were significantly higher than the vertical mirror scores for both words, *t* = 10.08, *df* = 382, *p* < .001, and non-words, *t* = 9.70, *df* = 382, *p* < .001. As vertical and horizontal mirror-sensitivity scores were highly correlated, *r* = .58, *t*(382) = 13.94, *p* < .001, the scores were averaged and a combined score was used for all analyses.

**Table 1.** Means and *SD*s of the horizontal and vertical mirror scores for word and non-word primes.

	Words		Non-words	
	*M*	*SD*	*M*	*SD*
Vertical score	0.003	0.199	−0.022	0.195
Horizontal score	0.236	0.249	0.224	0.290

*SD*: standard deviation.

#### Procedure

Due to the ongoing Covid-19 pandemic, the experiment was conducted as an online study. The experiment was performed using the Inquisit 5 Web app (Millisecond Software). Here, an API is downloaded and integrated in the browser of the local system. Thus, stimulus presentation and data collection were similar to a lab setting. Users were instructed to only do the experiment on a desktop or laptop computer and we discarded participants who worked on a mobile phone or tablet. However, we did not have control over variables such as display size and/or the refresh rate of the monitor. To standardise the layout and presentation of the stimuli, items were pre-captured and presented as images. Similarly, we asked participants at the beginning of the experiment to sit in a quiet room and prevent distractions for the time required to complete the experiment. We deliberately kept blocks rather short and included many breaks. Participants could decide on their own when to continue the experiment. Angele et al. (2022) have recently shown that it is possible to obtain reliable priming effects in online studies using similar conditions.

**Figure 2.**
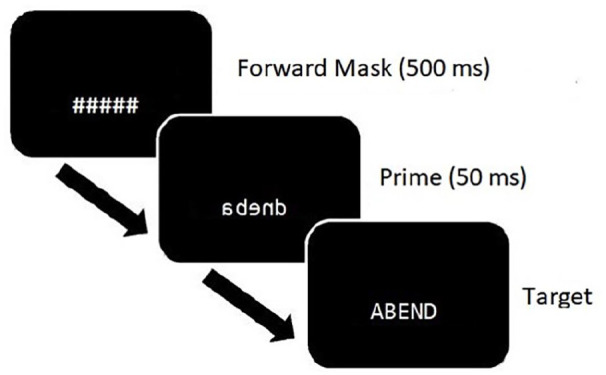
Schematic representation of a trial in the masked priming lexical decision task.

Participants performed a lexical decision task in which they were instructed to decide as fast and accurately as possible whether the items presented on the screen were words or non-words. There were four blocks which were separated by short pauses. In each block, participants responded to 48 words and 48 non-words. To respond, a key press of the letter K for words and the letter D for non-words was required. Response latencies and answers were recorded. All stimuli were presented in white, in the customised mirror-font with the font height set to 3.89% of the screen height. The background was black. Forward masks were created using hashes, and their length was identical to the length of the targets. Forward masks were presented for 500 ms. Immediately after this, the prime was presented for 50 ms. After that, the 50 ms target was then presented until a response was given (see Figure 2 for a schematic representation of a trial). Participants were not informed about the presence of the masked prime. In a survey that followed the experiment, none of the participants reported awareness of the masked primes. A different random order for the items was generated for each participant. Each participant received eight practice trials prior to the 384 experimental trials. The study was approved by the ethics committee of the University of Göttingen.

### Results

Results were analysed using (generalised) linear mixed-effects models using the lme4 package (Bates et al., 2015) for R (R Core Team, 2013). For response accuracy, a generalised linear mixed-effects model with a binomial link function was used. Correct log-transformed response times were analysed using a linear mixed-effects model. The data were trimmed in two stages. First, outliers were discarded by excluding all trials that were extremely fast (⩽300 ms) or slow (⩾3000 ms), which excluded 0.2% of all trials. In the second step, model criticism based on a simple model including only random intercepts for participants and items was used, excluding all data points exceeding 2.5 standard deviations. In this step, 2% of the trials were excluded.

We entered prime condition (4: identity vs. vertical vs. horizontal vs. control) and lexicality (2: word vs. non-word) as effect-coded fixed effects and random intercepts and random slopes for prime condition for both participants and items. In addition, control-target overlap and mirror-sensitivity were included as continuous variables in the model. Both variables were not centred because they have meaningful zero points. As a consequence, the overall effect of prime condition represents the priming effects for words without any overlap between prime and target and without reversible and symmetrical letters. The significance of the effects was evaluated using Wald tests and the ANOVA function of the car package using Type III model comparisons. Post hoc comparisons were conducted using the glht function in the multcomp package (Hothorn et al., 2008). The model mean RTs and accuracies are shown in Table 2, while the results from the linear mixed effect models are shown in Table 3.

**Table 2.** Experiment 1: mean model reaction times (milliseconds), accuracy (%), and priming effects to word and non-word targets (SEs in parentheses).

Words				Non-words				
Prime condition	Acc	∆	RT	∆	Acc	∆	RT	∆
Identity	99.4 (0.2)	2.7***	542 (13)	40***	98.2 (0.5)	0.5	647 (16)	15*
Vertical	98.8 (0.4)	2.1**	568 (14)	14*	97.5 (0.5)	0.2	652 (16)	11
Horizontal	96.9 (0.4)	0.2	583 (14)	−1	96.8 (0.4)	0.9	651 (16)	11
Control	96.7 (0.9)		582 (15)		97.7 (0.6)		662 (17)	

*Note*. Different letters indicates significant contrast.

*SE*: standard error; Δ: size of priming effect; RT: reaction times. * = *p* < .05, ** = *p* < .01, *** = *p* < .001.

**Table 3.** Analysis Experiment 1: results from linear mixed-effects models ( $\chi^{2}$ Wald tests) for word accuracy and RTs in Experiment 1.

	Accuracy		RT	
Effect (*df*)	χ^2^	P	χ^2^	P
Prime condition (3)	29.35	<.001***	52.16	<.001***
Lexicality (1)	2.85	.091	253.43	<.001***
Overlap (1)	0.19	.666	0.93	.335
Mirror-sensitivity (1)	0.31	.577	0.27	.606
Prime condition × Lexicality (3)	14.51	<.01**	24.83	<.001***
Prime condition × Overlap (3)	2.46	.483	0.33	.954
Lexicality × Overlap (1)	1.62	.203	1.61	.205
Prime Condition × Mirror-Sensitivity (3)	1.82	.610	2.56	.464
Lexicality × Mirror-Sensitivity (1)	8.58	<.01**	1.62	.203
Prime Condition × Lexicality × Overlap (3)	6.18	.103	1.48	.687
Prime Condition × Lexicality × Mirror-Sensitivity (3)	1.53	.676	0.41	.939

RT: reaction times.* = *p* < .05, ** = *p* < .01, *** = *p* < .001.

#### Reaction times

Results showed a main effect of prime condition which was qualified by a significant Prime Condition × Lexicality interaction. Post hoc contrasts showed that the simple main effect of prime condition was significant for words, χ^2^ = 69.25, *p* < .001, but not in non-words, $\chi^{2}$ = 6.01, *p* = .111. For words, there was a significant identity priming effect, Δ = 40 ms, *z* = 7.05, *p* < .001. The vertical mirror-priming effect was also significant, Δ = 14 ms, *z* = 2.53, *p* < .01, but the horizontal mirror-priming effect was not, Δ = −1 ms, *z* = −0.10, *p* = .918. The vertical and the horizontal priming effect were significantly different, *z* = 2.67, *p* < .01. The vertical and the identity priming effect were also significantly different, *z* = −5.014, *p* < .001.

In addition, the main effect of lexicality was also significant: Words (*M* = 569 ms, *SE* = 14 ms) were recognised Δ = 84 ms faster than non-words (*M* = 653 ms, *SE* = 16 ms).

#### Accuracy

There was a main effect of prime condition, which was qualified by a significant Prime Condition × Lexicality interaction. Post hoc contrasts showed that the simple main effect of prime condition was only significant in words, χ^2^ = 31.39, *p* < .001, but not in non-words, ${\text{χ}}^{2}$ = 5.40, *p* = .145. For words, there was a significant identity priming effect, ∆ = 2.7%, *z* = 4.59, *p* < .001, and a significant vertical priming effect, Δ = 2.1%, *z* = −3.29, *p*  < .01. There was no horizontal priming effect, Δ = 0.2%, *z* = 0.34, *p* = .732. The vertical and the horizontal priming effect were significantly different, *z* = 3.09, *p*  .01. The vertical priming effect did not differ significantly from the identity priming effect, *z* = 1.654, *p* = .098.

In addition, the Lexicality × Mirror-Sensitivity interaction was significant. Post hoc contrasts showed that the lexicality effect was only significant for primes with low mirror-sensitivity, *z* = 3.56, *p* <.001, but not for primes with high mirror-sensitivity, *z* = 0.28, *p* = .780.

### Discussion

The results from Experiment 1 are rather clear-cut. First, for words we found vertical, but not horizontal mirror-priming effects in both response time and accuracy. Vertical priming effects were, however, not as pronounced as the identity priming effects. For non-words, by contrast, mirror-priming effects were rather weak and inconsistent. Finally, both vertical and horizontal priming effects were not affected by words’ average mirror-sensitivity.

By and large, this pattern replicates important results from previous studies which found facilitatory priming effects for primes with vertically mirrored letters (Duñabeitia et al., 2011; Winskel & Perea, 2018). However, our study shows that mirror-priming effects can also be found when all, but not only single letters are mirrored. By contrast, horizontal mirror-priming effects are rather weak or non-existent.

This is the first masked priming study that has investigated mirror-priming effects for non-words. These were generally absent which seems to indicate that mirror-priming effects are confined to words and are lexical by nature (Perea et al., 2011).

Finally, our results show that average word mirror-sensitivity did not moderate mirror-priming effects which were facilitatory and equally strong for high- and low-confusable words. This finding does not contradict studies which found inhibitory priming effects for individual reversible letters (Perea et al., 2011; Soares et al., 2019). It shows, however, that these effects might be too subtle to be detected on the word level.

## Experiment 2

Overall, findings from Experiment 1 indicate that mirror-priming effects might be lexical and confined to vertical mirroring. Before accepting this conclusion, however, we conducted a second experiment with the same set of items as in Experiment 1 but using the same–different match task instead of lexical decision. As argued by Kinoshita and Norris (2012), the same-different task is particularly suited for studying early, pre-lexical processing stages of visual word processing. In contrast to the lexical decision task, participants do not have to decide whether the target is a word or a non-word, but whether it matches a previously displayed reference stimulus. This difference has a huge impact on the procedural level. To decide whether the target is a word or not, the reader does not have to identify *which* word the input is. Thus, the identification of a word’s individual component letters is not necessarily required by the lexical decision task. By contrast, the same-different match task requires the observer to recognise each individual letter because differences between the reference and the target stimulus can occur everywhere in the target. Visual, pre-lexical effects are thus generally boosted and priming effects are typically observed for both words and non-words (Kinoshita & Norris, 2012). We thus expected that mirror-priming effects would be generally larger and be observed for both words and non-words.

### Methods

#### Participants

We recruited a new sample of 42 adult participants via the participant recruiting system of the University of Göttingen for an online experiment. Participants had a mean age of M = 21.17 years (*SD* = 2.74; 33 females), were German native speakers, and had normal or corrected-to-normal vision.

#### Materials

The same materials as for Experiment 1 were used in Experiment 2. To create *different* trials for the same-different task, we added 192 reference words which were nouns from the DWDS (Geyken, 2007). All reference words had a normalised lemma frequency > 20 and had similar lemma frequencies as the target words, *t* = 1.01, *df* = 382, *p* = .843. Each reference-target word pair had the same length and did not comprise identical letters in the same position (i.e. armee-staat). Non-word references were created by substituting each vowel with a different vowel and each consonant with different consonant. Thus, as for words, reference and target non-words had the same length and did not comprise identical letters in the same position (i.e., arage-unoki).

To avoid repeating targets within the experiment, we used a Latin square design with eight item lists, such that each participant saw each target once in one of the four prime conditions and in either the same or the different condition. However, across participants, each target was presented in all conditions. Overall, each participant saw 192 words and 192 non-words with 24 words and 24 non-words in each of the four prime conditions in the same and different condition, respectively.

#### Procedure

**Figure 3.**
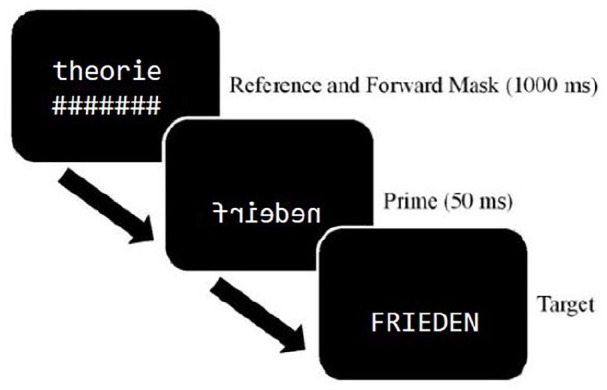
Schematic representation of a trial in the masked priming cross-case same–different match task.

Again, the experiment was conducted in an online study, using the same software and setting than as in Experiment 1. Participants performed a cross-case same–different match task in which they were presented with a pair of letter strings, one after another. Participants were instructed to decide as fast and accurately as possible whether the two letter strings were identical or different. Between reference and target word, a prime word was briefly displayed. In each block, participants responded to 48 words and 48 non-words. Participants responded by pressing the K (same) or D (different) key on the keyboard. Both response latencies and answers were recorded. Primes and references were always presented in lowercase, whereas targets were presented in uppercase. All stimuli were presented in white, in the customised mirror-font with the font height set to 3.89% of the screen height. The background was kept black. The reference was presented for 1000 ms, together with the forward mask that was presented for the same time in the line below the reference. After the 1000 ms, the reference and the mask vanished and the prime appeared at the location of the forward mask, which was presented for 50 ms. The target was then presented in this same location until a response was given (see Figure 3 for a schematic representation of a trial). Participants were not informed of the presence of the prime. In a survey that followed the experiment, none of the participants reported awareness of the masked primes. A different random order of items was generated for each participant. Each participant received eight practice trials prior to the 384 experimental trials. The study was approved by the Ethics Committee of the University of Göttingen.

#### Analyses

Data from same and different trials were analysed separately using (generalised) linear mixed-effects models. The cleaning procedure for correct response times was identical to Experiment 1, leading to an exclusion of 3.1% of responses overall. The models for the analyses were specified in the same way as in Experiment 1, with prime condition and lexicality as effect-coded fixed effects and random intercepts and random slopes for prime condition for participants and items. In addition, mirror-sensitivity and control-target overlap were included as continuous variables. Model mean RTs and accuracy are provided in Table 4 (Same-trials) and Table 5 (Different-trials), results from linear mixed-effects model are provided in Table 6 (Same-trials) and in Table 7 (Different-trials).

**Table 4.** Experiment 2: mean model reaction times (milliseconds), accuracy (%), and priming effects to word and non-word targets in same-responses (*SE*s in parentheses).

	Words					Non-words		
Prime condition	Acc	∆	RT	∆	Acc	∆	RT	∆
Identity	98.4 (0.5)	68***	446 (13)	78***	98.0 (1.3)	9.6***	482 (14)	75***
Vertical	97.6 (0.8)	60***	470 (13)	54***	96.9 (0.9)	8.5***	501 (14)	56***
Horizontal	97.1 (0.8)	55***	476 (14)	48***	95.3 (1.3)	6.9***	500 (14)	57***
Control	91.6 (1.7)		524 (13)		88.4 (2.0)		557 (14)	

*SE*: standard error; Δ: size of priming effect; RT: reaction times.* = *p* < .05, ** = *p* < .01, *** = *p* < .001.

**Table 5.** Experiment 2: mean model reaction times (milliseconds), accuracy (%), and priming effects to word and non-word targets in different-responses (*SE*s in parentheses).

	Words				Non-words			
Prime condition	Acc	∆	RT	∆	Acc	∆	RT	∆
Identity	98.7 (0.9)	0.2	523 (15)	−7	98.8 (0.4)	0.5	522 (15)	4
Vertical	99.2 (2.8)	0.7	514 (14)	2	97.8 (0.7)	0.5	530 (14)	−4
Horizontal	97.8 (1.5)	−0.7	526 (15)	−10	98.4 (0.5)	−0.3	525 (15)	1
Control	98.5 (1.0)		516 (13)		98.4 (0.5)		526 (13)	

*SE*: standard error; ∆: size of priming effect; RT: reaction times.* = *p* < .05, ** = *p* < .01, *** = *p* < .001.

**Table 6.** Experiment 2: results from linear mixed-effects models (χ^2^ Wald tests) for word RTs and accuracy for same-responses in Experiment 2.

	Accuracy		RT	
Effect (*df*)	χ^2^	P	χ^2^	P
Prime condition (3)	76.72	<.001***	218.10	<.001***
Lexicality (1)	5.15	.023*	76.32	<.001***
Overlap (1)	0.00	1.00	0.86	.353
Mirror-sensitivity (1)	0.18	.668	3.92	.048*
Prime condition × Lexicality (3)	0.56	.906	2.31	.511
Prime condition × Overlap (3)	14.20	.003**	8.28	.041*
Lexicality × Overlap (1)	0.00	.955	5.62	.018*
Prime Condition × Mirror-Sensitivity (3)	8.56	.036*	17.41	<.001***
Lexicality × Mirror-Sensitivity (1)	3.48	.062	9.10	.003**
Prime Condition × Lexicality × Overlap (3)	4.23	.238	2.31	.511
Prime condition × Lexicality × Mirror-sensitivity (3)	0.94	.817	2.43	.488

RT: reaction times.* = *p* < .05, ** = *p* < .01, *** = *p* < .001.

**Table 7.** Experiment 2: results from linear mixed-effects models (χ^2^ Wald tests) for word RTs and accuracy for different-responses in Experiment 2.

	Accuracy			RT
Effect (*df*)	χ^2^	P	χ^2^	P
Prime condition (3)	7.58	.056	0.9	.882
Lexicality (1)	0.00	.974	3.07	.080
Overlap (1)	0.00	.955	0.32	.574
Mirror-sensitivity (1)	0.02	.891	2.51	.113
Prime condition × Lexicality (3)	0.70	.874	5.02	.170
Prime condition × Overlap (3)	3.50	.321	3.01	.390
Lexicality × Overlap (1)	1.00	.318	0.08	.774
Prime Condition × Mirror-Sensitivity (3)	1.53	.676	3.46	.327
Lexicality × Mirror-Sensitivity (1)	0.12	.732	0.17	.680
Prime Condition × Lexicality × Overlap (3)	2.67	.445	8.67	.034*
Prime Condition × Lexicality × Mirror-sensitivity (3)	0.53	.913	0.93	.818

RT: reaction times.* = *p* < .05, ** = *p* < .01, *** = *p* < .001.

### Results

#### Reaction times

For same-trials, the main effect of prime condition as well as the Prime Condition × Mirror-Sensitivity, the Lexicality × Mirror-Sensitivity and the Prime-Condition × Overlap Score interactions were significant.

Post hoc contrasts showed a substantial identity priming effect, Δ = 76 ms, *z* = 14.39, *p* < .001. In addition, there were mirror-priming effects for both vertical, ∆ = 55 ms, *z* = 10.93, *p* < .001, and horizontal mirror-primes, Δ = 52 ms, *z* = 9.68, *p* < .001. The size of vertical and horizontal priming effect did not differ from each other, *z* = 0.64, *p* = .521. Both the horizontal mirror-priming effect, *z* = 5.468, *p* <.001, and the vertical mirror-priming effect, *z* = 4.867, *p* <.001, differed significantly from the identity priming effect.

The Prime Condition × Mirror-Sensitivity interaction indicated that priming effects were reduced for words with high mirror-sensitivity: identity priming, ∆ = 63 ms, *z* = 11.87, *p* < < << < .001, vertical mirror-priming, ∆ = 45 ms, *z* = 8.91, *p* < << < .001, and horizontal mirror-priming, Δ = 45 ms, *z* = 8.91, *p* < << <.001.

The Prime Condition × Overlap Score interaction indicated that priming effects were weaker for trials with higher overlap between prime and target: identity priming, Δ = 68 ms, *z* = 14.35, *p* <.001, vertical mirror-priming, Δ = 49 ms, *z* = 11.04, *p* <.001, and horizontal mirror-priming, Δ = 43 ms, *z* = 8.82, *p* <.001.

In addition, the main effects of lexicality and mirror-sensitivity as well as the Lexicality × Mirror-Sensitivity and the Lexicality × Overlap interactions were significant. RTs for words, *M* = 478, *SE* = 12, were shorter than for non-words, *M* = 509, *SE* = 13. The simple main effect of mirror-sensitivity was not significant in words, *b* = −0.014, *z* = 0.88, *p* = .483, but in non-words, *b* = 0.066, *z* = 3.72, *p* < << < .001. By contrast, the simple main effect of overlap was only significant in words, *b* = −0.013, *z* = −2.41, *p* = .016, but not in non-words, *b* = 0.006, *z* = 0.99, *p* = .323.

In different trials, the Prime Condition × Lexicality × Overlap interaction just reached significance. Only the identity priming was significant, *z* = −2.41, *p* = .016, but in the opposite direction, Δ = −13 ms.

#### Accuracy

For same-trials, the main effect of prime condition was significant as well as the Prime Condition × Mirror-Sensitivity and Prime Condition × Overlap interactions.

Post hoc contrasts showed that the identity priming effect, Δ = 8.1%, *z* = 7.26, *p* < << < .001; the vertical mirror-priming effect, ∆ = 7.1%, *z* = 6.20, *p* <.001; and the horizontal mirror-priming effect Δ = 6.2%, *z* = 5.85, *p* < << < .001, were significant. Vertical and horizontal mirror-priming effects did not differ significantly from each other, *z* = 1.31, *p* = .190. The horizontal mirror-priming effect differed significantly from the identity priming effect, *z* = −3.321, *p* <.001, whereas the vertical mirror-priming effect did not, *z* = −1.851, *p* = .064.

The Prime Condition × Mirror-Sensitivity interaction indicated that priming effects were smaller for primes with high mirror-sensitivity: identity priming, ∆ = 5.8%, *z* = 5.56, *p* < << < .001; vertical mirror-priming, Δ = 5.4%, *z* = 5.11, *p* <.001; horizontal mirror-priming, Δ = 4.3%, *z* = 4.40, *p* < << < .001.

The Prime Condition × Overlap interaction showed that priming effects were smaller for primes with higher overlap between control primes and target: identity priming, Δ = 5.4%, *z* = 5.6, *p* <.001; vertical mirror-priming, Δ = 5.0%, *z* = 5.33, *p* <.001; horizontal mirror-priming, Δ = 4.0%, *z* = 4.71, *p* <.001.

For different trials, no effects were significant.

### Discussion

Again, the effects reported in Experiment 2 are rather straightforward, but different from the effects found in Experiment 1. First, we found substantial and equally strong mirror-priming effects in the vertical and the horizontal priming condition which, however, were not as large as the identity priming effects. Second, we observed similar priming effects for words and non-words. Indeed, priming effects for words and non-words were nearly identical, differing only by a few milliseconds (see Table 4). Finally, we saw that mirror-priming effects were moderated by letter mirror-sensitivity. Generally, priming effects were smaller for high- than for low-mirror-sensitive words.

Overall, the results of Experiment 2 indicate that mirror-priming effects have a pre-lexical orthographic locus and that they occur for both vertically and horizontally mirrored letters. This finding directly extends the findings reported by Duñabeitia et al. (2011) and Winskel and Perea (2018) who, however, only investigated vertical mirror-priming. We also found that mirror-priming effects were stronger for low- compared with high-mirror-sensitive non-words. This is in line with the findings of Perea et al. (2011) and Soares et al. (2019), who showed that mirroring of reversible letters produces interference effects which might lead to decreased mirror-priming effects in the high mirror-sensitivity condition.

## General discussion

In the present study, we conducted two experiments to examine how letter mirroring impacts visual word recognition and, in particular, the early stages of orthographic processing. We used a masked priming paradigm using primes with vertically and horizontally mirrored letters. Task was varied between experiments: In Experiment 1, we used a lexical decision task whereas in Experiment 2 we used a cross-case same-different match task. In the lexical decision task, we found vertical mirror-priming effects for both response times and response accuracy, but only for words. In the same-different task, by contrast, we found both vertical and horizontal mirror-priming effects for both words and non-words. In addition, mirror-priming effects were moderated by mirror-sensitivity, with weaker effects for words comprising more reversible letters.

Thus, a first general conclusion from our study is that mirror-priming effects vary with task. As pointed out by Kinoshita and Norris (2012), different tasks tap into different processes of the word recognition process. The same-different match task is suitable for examining the early, pre-lexical processes of word recognition, whereas the lexical decision task primarily reflects lexical priming. Hence, any potential mirror-priming effects in a lexical decision task are likely to be modulated by lexical access, and thus, weak mirror-priming effects at the feature and letter level may be obscured by higher-level feedback from the word level.

Second, we found that mirror-priming effects were less pronounced than the identity priming effect (graded mirror-priming effects). It is thus unlikely that mirror-image generalisation is completely preserved. Rather, there is a residual mirror-priming effect which leads to the observed facilitatory effects of non-reversible mirror letters. Although these facilitatory effects have a pre-lexical orthographic locus, they seem to be influenced by top-down feedback from the word level as suggested by differences due to the task (in Experiment 1), or due to the stimulus material (in Experiment 2).

However, the observed differences between the two tasks might also be interpreted within the levels-of-processing framework (Roediger & Blaxton, 1987). Specifically, the lexical decision task (which involves lexical processing) might induce deeper and more analytical processing, while letters in the same–different task (which does not involve lexical processing) are processed more holistically (Bamber, 1972; Ventura et al., 2021).

Next to differences between tasks, it is likely that other aspects of the task (such as prime duration or response categories) will also affect the observed pattern of results (Lachmann & van Leeuwen, 2008).

Third, we extend previous research by showing that vertically (left–right) and horizontally (up–down) mirrored letters evoke equally strong priming effects, indicating that during early feature- and letter processing, mirror-priming effects operate both vertically and horizontally. This suggests that at the initial stages of processing, letter features are activated irrespective of their left–right or up–down orientation, following general visual principles of symmetry-generalisation (Lachmann, 2002).

And finally, we found stronger mirror-priming effects for low than for high mirror-sensitive non-words. This shows that priming effects are reduced when targets comprise reversible letters. This reduction is presumably driven by interference effects at the letter level caused by the simultaneous activation of competing letter representations (Perea et al., 2011; Soares et al., 2019). This finding also shows that the tendency to implicitly mirror letters is not selectively suppressed for reversible letters and that the same general principles of the visual processing apply to all letters (Duñabeitia et al., 2011; Poldrack et al., 1998).

However, as suggested by Fernandes et al. (2022) and Brossette et al. (2022), another alternative explanation that could account for the observed facilitatory effects of mirrored letters is visual similarity. Mirror-priming effects could be due to a partial activation of the feature detectors for the canonical letter which in turn activate the corresponding abstract letter representation. In fact, we are not able to distinguish between visual similarity and mirror-priming effects but the alternative explanation provides a clear, testable prediction. If mirror-priming effects are merely due to visual similarity, then words with letters that are rotated by 180° should evoke equally strong priming effects as words with mirrored letters.

Overall, the pattern of findings is consistent with the assumptions of the Interactive Activation Model (IAM) (McClelland & Rumelhart, 1981; Rumelhart & McClelland, 1982). The IAM assumes that a letter node is activated in response to the perception of features which match those of a letter’s abstract representation. Given the tendency of the visual system to mirror-generalise visual input, non-reversible letters (e.g., a, s, c) immediately activate the corresponding abstract letter representation because there is no inhibitory link between a non-reversible letter and its mirror-image counterpart. Reversible letters (b, d, p, q, n, u), by contrast, activate a competing letter representation which produces inhibitory effects at the letter level.

Similarly, the Bayesian Reader Model (Norris, 2006; Norris & Kinoshita, 2008, 2012) assumes that readers integrate information through the computation of conditional probabilities based on Bayes theorem, and the output of this computation is the likelihood of a specific hypothesis given the evidence. The evidence corresponds to the visual input which the reader perceives. In contrast to the IAM, the Bayesian Reader assumes that both the prime and the target are processed as a single object. Thus, both the uppercase letter in the target and the lowercase letter in the prime provide evidence towards the hypothesis that the target contains a particular abstract letter representation in a specific location. Mirror-primes introduce less uncertainty to recognition than control primes because mirror-reversals of letters comprise the same features and have the same overall visual shape as letters in their normal position. However, the presence of reversible letters within the prime increases the likelihood that a different letter is present in a specific location within target. This leads to an increased level of noise for reversible compared with non-reversible letters which in turn decreases mirror-priming effects in high-mirror-sensitive words.

So, how are mirror-priming effects generated in such models? First, results indicate that at the level of features, the coding scheme is highly flexible because it seems to activate individual features regardless of the reader’s viewpoint (Perea et al., 2011). There are several distinctive features that have been identified for the letters of the Latin alphabet. These include terminations, straight lines, curved lines, and oblique lines, as well as intersections (Briggs & Hocevar, 1975; Fiset et al., 2008; Gibson, 1969). For the distinction of oblique lines, the direction of mirroring is irrelevant because/ equals \ irrespective of the direction of mirroring. By contrast, curved lines have, despite their shape, another additional relevant characteristic which is the opening direction (either up–down or left–right) of their arc. If letter features are activated regardless of one’s viewpoint, then the feature 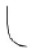 would partially activate 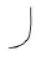, and 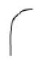, whereas straight vertical and horizontal lines do not change their orientation when mirrored either way. In this example, the horizontal mirror-prime of the letter < f > (e.g., 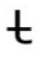) likely activates the two competing abstract letter representations f/F and t/T, whereas the vertical mirror-prime (e.g., 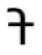) activates only the abstract letter representation f/F. It has to be noted, though, that the current implementation of the IAM only includes uppercase letters and, thus, a set of lowercase letters would need to be added (Perea et al., 2011).

In conclusion, we extended previous masked priming research (Duñabeitia et al., 2011; Perea et al., 2011; Soares et al., 2019, 2021; Winskel & Perea, 2018) by investigating the effects of both vertical and horizontal mirror-primes and in both words and non-words in a lexical decision and a same–different match task. We found vertical and horizontal mirror-priming effects for both words and non-words in the same-different task. This indicates that the observed priming effects operate at the feature and letter level of processing and that they are likely to be influenced by lexical dynamics.
